# Five Years of Acute Stroke Unit Care: Comparing ASU and Non-ASU Admissions and Allied Health Involvement

**DOI:** 10.1155/2014/798258

**Published:** 2014-03-03

**Authors:** Isobel J. Hubbard, Malcolm Evans, Sarah McMullen-Roach, Jodie Marquez, Mark W. Parsons

**Affiliations:** ^1^School of Medicine and Public Health, University of Newcastle, Level 4 Hunter Medical Research Institute, 1 Kookaburra Circuit, New Lambton Heights, NSW 2305, Australia; ^2^Neurology Department, John Hunter Hospital, Hunter New England Health, Locked Bag 1, Hunter Region Mail Centre, NSW 2301, Australia; ^3^Discipline of Occupational Therapy, School of Health Sciences, Faculty of Health, University Drive, University of Newcastle, Callaghan NSW 2308, Australia; ^4^Discipline of Physiotherapy, School of Health Sciences, Faculty of Health, University Drive, Callaghan, NSW2308, Australia; ^5^School of Medicine and Public Health, University of Newcastle, Hunter New England Local Health District, Callaghan NSW 2308, Australia

## Abstract

*Background.* Evidence indicates that Stroke Units decrease mortality and morbidity. An Acute Stroke Unit (ASU) provides specialised, hyperacute care and thrombolysis. John Hunter Hospital, Australia, admits 500 stroke patients each year and has a 4-bed ASU. *Aims.* This study investigated hospital admissions over a 5-year period of all strokes patients and of all patients admitted to the 4-bed ASU and the involvement of allied health professionals. *Methods.* The study retrospectively audited 5-year data from all stroke patients admitted to John Hunter Hospital (*n* = 2525) and from nonstroke patients admitted to the ASU (*n* = 826). The study's primary outcomes were admission rates, length of stay (days), and allied health involvement. *Results.* Over 5 years, 47% of stroke patients were admitted to the ASU. More male stroke patients were admitted to the ASU (chi^2^ = 5.81; *P* = 0.016). There was a trend over time towards parity between the number of stroke and nonstroke patients admitted to the ASU. When compared to those admitted elsewhere, ASU stroke patients had a longer length of stay (*z* = −8.233; *P* = 0.0000) and were more likely to receive allied healthcare. *Conclusion.* This is the first study to report 5 years of ASU admissions. Acute Stroke Units may benefit from a review of the healthcare provided to all stroke patients. The trends over time with respect to the utilisation of the John Hunter Hospitall's ASU have resulted in a review of the hospitall's Stroke Unit and allied healthcare.

## 1. Introduction 

In Australia, stroke is the leading cause of long-term disability and the second most common cause of death [1]. The two most effective interventions after stroke are organised Stroke Unit Care [2] and thrombolysis [3]. The National Stroke Foundation's clinical guidelines identified Stroke Unit Care as the most important “intervention” on offer to Australians affected by stroke [4], with evidence indicating that it increases independence, survival, and rates of living at home at 12 months by 20% [5–9]. Stroke Unit Care improves functional outcomes and decreases length of stay when compared to patients admitted elsewhere [3, 10, 11]. It is characterised by geographically designated beds and an educated and enthused multiprofessional team [6]. The Acute Stroke Unit (ASU) was introduced to provide specialised, hyperacute care and thrombolysis. Thrombolysis was first licensed in 1996 for use in stroke in the United States of America [12], and despite its efficacy in those with an ischemic stroke, evidence indicates that it is still only available to a limited number of patients. For the purposes of this paper, an ASU will describe a unit which offers to those with a very recent stroke a short-term acute admission and hyperacute interventions such as poststroke thrombolysis. The resources required to support an ASU have meant that they are usually located in larger, tertiary hospitals. To date there are no longitudinal studies reporting ASU utilisation over time.

This study is based on data from the John Hunter Hospital, a regional hospital in New South Wales, Australia. It is a national leader in poststroke thrombolysis, offering it to approximately 20% of all ischaemic strokes patients [13]. The hospital has 800 beds and admits around 500 stroke patients each year. It has a 4-bed ASU that was established in 2003, and these are the only stroke-designated beds in the hospital. The ASU is located within a 16-bed Neurology Unit that has an on-call stroke response team, access to onsite neuroimaging, a specialised multiprofessional team, an emergency department protocol for rapid triage, a license to administer thrombolysis, and access to intensive care and subacute services. National audits [14, 15] identified a decrease over time in the number of stroke patients being admitted to the ASU and a decrease in the early involvement of allied health professionals. These changes, which were of concern to those in the Neurology Unit, provided the “driver” for this investigation.

## 2. Aims

This study will investigate changes in ASU utilisation with particular focus on the admission of acute stroke patients. It aims to answer the following questions: How many strokes patients and nonstroke patients were admitted to the 4-bed ASU and did this change over time? What was the involvement of allied health professionals in acute stroke patients? Were there discrepancies between the length of stay and allied healthcare provided to stroke patients admitted to the ASU and those admitted elsewhere (non-ASU)?

## 3. Method

This study retrospectively audited health data obtained between January 2005 and December 2009. Data included all John Hunter Hospital admissions with an International Classification of Diagnosis (ICD-10) of stroke at discharge and all nonstroke admissions to the ASU. The study received ethical approval from the Human Research Ethics committees of the hospital and the University of Newcastle. The study did not actively recruit participants but utilised routinely collected hospital data which was deidentified on receipt. The John Hunter Hospital is situated in regional New South Wales but has one of the largest intakes of stroke patients in Australia. It admits approximately 500 stroke patients each year who are primarily residents of the Newcastle, Lake Macquarie, and Hunter Valley regions. Patients can take up to 90 minutes to reach the hospital by car, but most patients would be travelling for around 15 to 20 minutes. The region's primary employers are the Hunter New England Local Health District and the University of Newcastle. Stroke patients are usually only in the John Hunter Hospital during the acute phase of their admission. If they require ongoing rehabilitation, they are transferred to Rankin Park Hospital or to a private hospital.

### 3.1. Inclusion/Exclusion

Patients were included if they had one of five ICD-10 discharge diagnoses for ischaemic stroke, haemorrhagic stroke, and/or transient ischaemic attack [16]. Those with a discharge diagnosis of subarachnoid or subdural haemorrhage were excluded. Patients not admitted to the ASU, but still diagnosed with stroke, were excluded if the sole purpose of the admission was waiting for a rehabilitation bed or placement in a supported accommodation facility. Patients were excluded if their admission was secondary to a previous acute admission and/or if the admission category was rehabilitation, maintenance care, or geriatric evaluation and management (*n* = 787). Patients were also excluded if they were under 18 years of age (*n* = 25).

The study's primary outcomes were admission rates, length of stay (days), and allied health involvement. STATA IC10 (STATA Corporation, TX) was used for all statistical analysis. To detect between-group differences in ASU and non-ASU stroke patients, a two-sample Wilcoxon rank-sum test was applied for age and length of stay and Pearson's chi squared test was applied for gender. Correlation coefficients were used to measure the odds ratios in allied health involvement.

## 4. Results

### 4.1. Admission of Stroke Patients over Time

**Figure 1:**
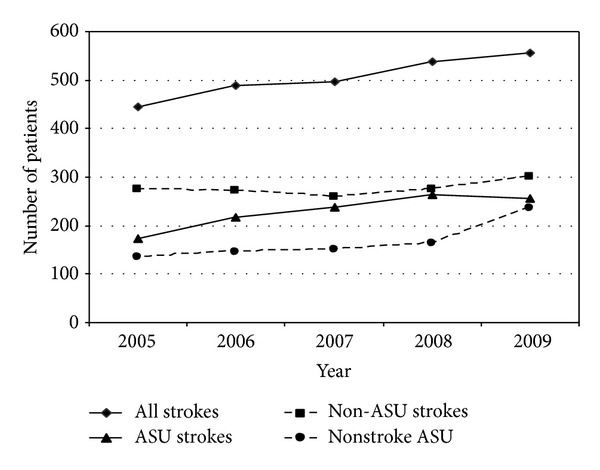
Comparing the number of patients admitted to the John Hunter Hospital who were diagnosed with stroke (all strokes), with stroke and admitted to the ASU (ASU strokes), and with stroke and admitted elsewhere (non-ASU strokes) and patients not diagnosed with stroke but admitted to the ASU (nonstroke ASU).

The study included 2525 stroke patients and 826 nonstroke patients (*N* = 3351). Over the 5-year period, 47% of patients (*n* = 1181) with stroke were admitted to the ASU and 53% (*n* = 1344) were admitted elsewhere (non-ASU). Their mean age was 72.2 years (SD14.91) and there was no significant difference between the mean age of ASU and non-ASU stroke patients (*z* = 1.28; *P* = 0.21). Over 5 years the number of stroke patients admitted to the hospital increased alongside the number of stroke patients admitted to the ASU (Figure 1). When compared to stroke patients admitted elsewhere, more men than women were admitted to the ASU (chi² = 5.81; *P* = 0.016). Also, the length of stay in stroke patients admitted to the ASU was longer (*z* = −8.233; *P* = 0.0000) than stroke patients admitted elsewhere.

**Figure 2:**
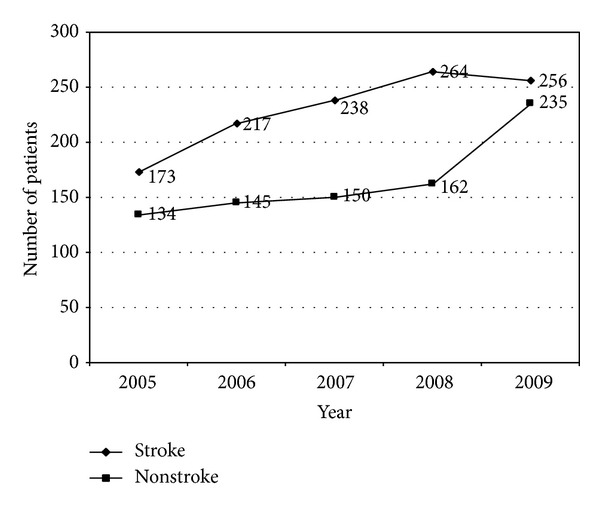
Number of patients admitted to the ASU: comparing stroke patients and nonstroke patients.

Over 5 years the number of stroke and nonstroke patients admitted to the ASU increased (Figure 2). Results indicated that, in each consecutive year, nonstroke patients accounted for at least 40% of all ASU admissions. Although the number of nonstroke patients never exceeded the number of stroke patients, in 2009, only 52% of patients in the ASU were diagnosed with stroke.

### 4.2. Allied Health Involvement in Stroke Patient Care

**Figure 3:**
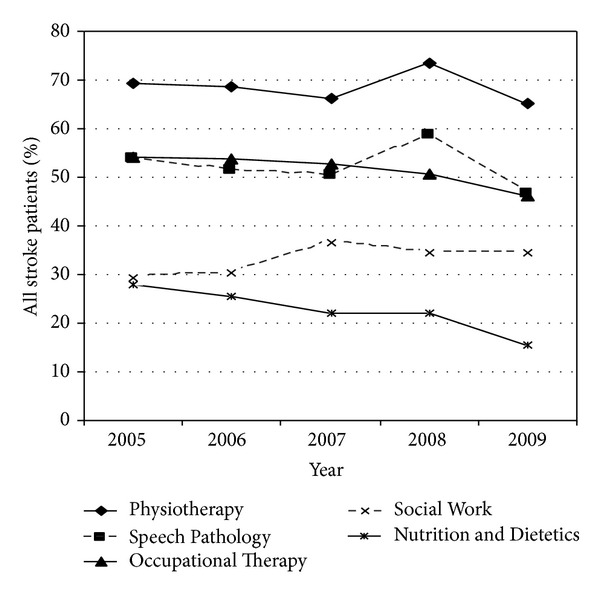
Percentage of all stroke patients who received care from allied health professionals.

Irrespective of whether or not stroke patients were admitted to the ASU, two in every three patients received Physiotherapy during their acute admission to the John Hunter Hospital, and one in every two received Occupational Therapy and Speech Pathology (Figure 3).

Over the 5-year study period, the number of ASU stroke patients that received Physiotherapy, Speech Pathology, Occupational Therapy, and Social Work increased over time but the percentage remained relatively stable (Table 1). In contrast, the percentage and number of ASU stroke patients that received healthcare from “Nutrition & Dietetics” halved. Over 5 years the percentage of non-ASU stroke patients that received care from allied health professionals decreased by 10% for Physiotherapy, 18% for Speech Pathology, and 12% for Occupational Therapy.

When compared to stroke patients admitted elsewhere, those admitted to the ASU were 30% more likely to receive Physiotherapy, 50% more likely to receive Speech Pathology, and 40% more likely to receive Occupational Therapy (Table 2).

### 4.3. Summary

Over the 5-year study period the majority of patients with acute stroke were not admitted to the ASU and in turn not admitted to a hospital bed that was designated for patients with stroke. During this time, between 40 and 50% of all ASU admissions were allocated to patients who were not diagnosed with stroke. Stroke patients admitted to the ASU had a longer length of stay and more of them were men. Stroke patients admitted to the ASU were more likely to receive allied healthcare. This involvement remained relatively consistent for stroke patients admitted to the ASU but was decreasing for those admitted elsewhere. Irrespective of whether or not they were admitted to the ASU, two-thirds of acute stroke patients received Physiotherapy and around half received Occupational Therapy and Speech Pathology.

## 5. Discussion

### 5.1. ASU Stroke and Nonstroke Admissions

This is the first study to identify concerns about the utilisation of an ASU over time. Although there was an increase in the number of stroke patients admitted to the John Hunter Hospital, there appeared to be ongoing pressure on the hospital's ASU to admit nonstroke patients. Over 5 years, nearly two ASU beds and two ASU admission days were relinquished to nonstroke patients. The ASU is located on a Neurology and Neurosurgical ward and there is persistent pressure to allocate the ASU beds to other high-acuity neurological patients, for example, those with seizures. Considering that the ASU holds the hospital's only stroke-designated beds, this is essentially denying between 40 and 50% of all acute stroke patients' access to healthcare that complies with the nationally-agreed clinical guidelines [4, 17]. It could also be decreasing the rates of independence, survival, and discharge home in this group of patients [5–9]. This evidence is therefore of concern.

### 5.2. Differences between ASU and Non-ASU Stroke Patients

This study found that a stroke patient's age did not impact whether or not they were admitted to the ASU, but, in contrast, a stroke patient's gender did. Evidence indicates that both age and gender can impact patient bed allocation as health services attempt to maximise resource efficiencies [18, 19]. The finding of a bias towards male patients with acute stroke is in contrast to the findings of a Canadian investigation which examined the influence of gender in acute stroke care (*n* = 3323) over a 12-month period [20]. Equity of health care is a central tenet of the World Health Organisation's platform [21]. The reason why more men than women were admitted to the hospital's ASU is difficult to explain and needs further investigation. The increase in total numbers of patients being admitted to the ASU underpins the decrease over time in a stroke patient's length of stay. The findings that admission to the ASU increased a stroke patient's length of stay are in conflict with the findings that admission to a Stroke Unit decreases length of stay [9, 22, 23]. It is also in conflict with Chen, McClaran, and Buchan [24] who examined the impact of an ASU in the United Kingdom and found a steady and significant reduction in the length of stay in stroke patients which did not adversely affect patient outcomes. This may identify differences in Acute Stroke Units and/or differences in the type of stroke patients admitted to an ASU.

### 5.3. Involvement of Allied Health Professionals

When compared to stroke patients not admitted to the ASU, those admitted to the ASU were more likely to receive Physiotherapy, Occupational Therapy, and Speech Pathology. Although there were no differences related to the involvement of Nutrition and Dietetic professionals, their involvement retracted the most over the 5-year period. The finding that stroke patients not admitted to the ASU were less likely to receive Physiotherapy, Occupational Therapy, and Speech Pathology is of particular concern but supports the significance of Stroke Unit Care. Clinical guidelines consistently recommend a multiprofessional team approach to stroke management [25] and this approach was also a significant variable in the evidence supporting the effectiveness of Stroke Unit Care. Therefore, any indication of a shift away from evidence-based, stroke management is of concern and it can potentially adversely impact recovery outcomes in stroke patients [2, 8, 26–28].

### 5.4. Strengths and Limitations

The ASU model of care was introduced almost a decade ago and this study is the first to examine utilisation over 5 years and the first to investigate allied health involvement. However, its findings are specific to an Australian ASU and although having national significance should not necessarily be generalised to Acute Stroke Units elsewhere. This study's findings have generated a review of the John Hunter Hospital's ASU and consideration is being given to redesigning the acute care that this hospital provides to stroke patients. Further research is needed to investigate differences in Acute Stroke Units and their admission protocols and allied health involvement as this may be impacting length of stay and/or recovery outcomes.

## 6. Conclusion

This study is the first to identify longitudinal trends that may be associated with an Acute Stroke Unit. As a result of these findings that demonstrated that stroke patients were being potentially denied access to Stroke Unit Care, the hospital's Stroke team has submitted a proposal to either establish a Comprehensive Stroke Unit or establish an 8-bed modified Stroke Unit that includes a 4-bed Hyperacute Stroke Unit. To reclaim the efficiencies of Stroke Unit Care for all acute stroke patients, the authors recommend Acute Stroke Units review utilisation and if necessary implement changes to adjust for any inequities identified.

## Figures and Tables

**Table 1:** Percentage (number) of patients with stroke that received healthcare from Physiotherapy, Speech Pathology, Occupational Therapy, Social Work, and Nutrition and Dietetic per year: ASU versus non-ASU.

Stroke patients	2005	2006	2007	2008	2009
ASU patients	**100% (446)**	**100% (488)**	**100% (497)**	**100% (537)**	**100% (557)**
Physiotherapy	31% (137)	36% (177)	37% (185)	41% (222)	37% (204)
Speech Pathology	27% (120)	31% (150)	32% (158)	37% (200)	33% (183)
Occupational Therapy	27% (119)	30% (145)	31% (155)	31% (165)	28% (158)
Social Work	10% (44)	11% (55)	18% (88)	15% (82)	15% (83)
Nutrition and Dietetics	13% (58)	11% (55)	13% (63)	10% (56)	6% (33)
Non-ASU patients	**100% (273)**	**100% (271)**	**100% (259)**	**100% (273)**	**100% (301)**
Physiotherapy	63% (172)	58% (158)	56% (145)	63% (173)	53% (159)
Speech Pathology	44% (120)	37% (101)	36% (92)	42% (114)	26% (78)
Occupational Therapy	45% (123)	44% (118)	37% (97)	39% (107)	33% (99)
Social Work	32% (87)	34% (93)	36% (93)	38% (104)	37% (110)
Nutrition and Dietetics	25% (67)	25% (69)	18% (47)	23% (62)	18% (53)

**Table 2:** Allied health involvement: odds ratios ASU versus non-ASU stroke patients.

Allied health involvement	Risk ratio	*Z *	CI	*P* value
Physiotherapy	0.73	−11.83	0.69 to 0.77	*P* < 0.001
Speech Pathology	0.52	−16.22	0.48 to 0.56	*P* < 0.001
Occupational Therapy	0.61	−12.42	0.56 to 0.66	*P* < 0.001
Social Work	2.55	0.67	1.03 to 1.27	*P* = 0.11
Nutrition and Dietetics	0.91	−1.29	0.79 to 1.05	*P* = 0.198
